# The association between quality of life and subjective wellbeing among older adults based on canonical correlation analysis

**DOI:** 10.3389/fpubh.2023.1235276

**Published:** 2023-09-19

**Authors:** Huanting Liu, Qihui Gan, Jianfeng Tan, Xiaoyuan Sun, Yuxi Liu, Chonghua Wan

**Affiliations:** ^1^Key Laboratory for Quality of Life and Psychological Assessment and Intervention, Research Center for Quality of Life and Applied Psychology, School of Humanities and Management, Guangdong Medical University, Dongguan, China; ^2^Institute of Health Law and Policy, Guangdong Medical University, Dongguan, China

**Keywords:** happiness, subjective wellbeing, quality of life, older adult, canonical correlation

## Abstract

**Introduction:**

The study explored the relationship between subjective well-being and the quality of life among older adults. It highlights the importance of understanding how these factors are interconnected in the context of an aging population.

**Methods:**

Descriptive statistics were used to analyze the scores of general demographic characteristics, subjective wellbeing and quality of life. Simple correlation analysis and canonical correlation analysis were employed to analyze the relationship between subjective wellbeing and quality of life among older adults.

**Results:**

Data from 892 older adults were collected. Canonical correlation analysis revealed four pairs of canonical variables, with the first four pairs of canonical correlation coefficients all being statistically significant (0.695, 0.179, 0.147, 0.121) (*p* < 0.05), and the first pair of canonical variables explaining 93.03% of the information content. From the canonical loading coefficients, Vitality and mental health contributed the most to the quality of life (U1) canonical variable. The canonical variable V1, which corresponded to subjective wellbeing, was reflected by a combination of positive affect, negative affect, positive experience and negative experience. X1 (physical functioning), X2 (role-physical), X3 (bodily pain), X4 (general health), X5 (vitality), X6 (social functioning), X7 (role-emotional) and X8 (mental health) were positively correlated with Y1 (positive affect) and Y3 (positive experience), negatively correlated with Y2 (negative affect) and Y4 (negative experience). Cross-loadings revealed that physical functioning, bodily pain, general health, vitality, social functioning and mental health were the main factors reflecting the subjective wellbeing of older adults.

**Discussion:**

As quality of life among older adults was highly correlated with subjective wellbeing, appropriate measures should be taken to account for individual characteristics of older adults, and various factors should be integrated to improve their subjective wellbeing.

## 1. Introduction

Population aging is a common social phenomenon throughout the contemporary world, including in China, whose population is gradually aging. According to the National Bureau of Statistics of China, by 2020, 13.5% of China’s total population was aged 65 or older. Due to their age, older adults face problems such as declining physiological functions, which in turn lead to a diminished sense of wellbeing. This is problematic, as wellbeing – especially among older adults – is vital for improving quality of life, promoting mental health and coping with the needs of an aging population (1).

The term subjective wellbeing includes affective states, the self- evaluations of quality of life and individual events and circumstances, including residence type (2). Subjective wellbeing was often used to assess overall quality of life (3, 4). Studies have shown that physical health status, mental health problems and subjective wellbeing among older adults were negatively correlated (5). Subjective wellbeing was influenced not only by health but also by lifestyle factors unrelated to health (6). It was also been found that the level of subjective wellbeing was influenced by other factors, such as personality (7), culture (8), interpersonal interaction (9), marital status (10), and economic factors (11). In recent years, there has been a growing recognition of the importance of subjective wellbeing and its various determinants, particularly among the older adult population. As societies continue to age, the wellbeing of older individuals becomes a significant area of concern, demanding a shift in focus toward their holistic experiences. Recent studies have focused on factors affecting subjective wellbeing among older adults, mainly demographic differences (12), rural residency (13), physical exercise (14), social support (15), and environmental conditions (16). There is a growing body of research demonstrating the relationship between social capital and wellbeing, with social capital positively affecting subjective wellbeing. Zhang (17) showed that social capital is a favorable predictor of wellbeing. Furthermore, the connections, trust, and support derived from social relationships, were closely intertwined with the wellbeing of older adults (18). As social dynamics evolve over time, older adults can experience changes in their social networks, potentially affecting their health and wellbeing (19, 20). Liu et al. (21) found that the neighborhood environment had a significant impact on older adults’ subjective wellbeing. Participation in social activities (22) and volunteering (23) also had a positive impact on wellbeing. Recognizing the significance of social capital sheds light on the ways in which communities and institutions can foster an environment that nurtures positive interactions and meaningful relationships among older individuals.

These factors were fundamentally related to quality of life (24). The WHO defined quality of life (25) as the experience of individuals from different cultures and value systems concerning their life goals, expectations, standards, and state of life in relation to the things they cared about. This concept encompasses physical health, mental health, independence, social relationships, personal beliefs and interactions with the surrounding environment. It has been demonstrated that quality of life among older adults is both positively and negatively associated with the construction of subjective wellbeing (26). In one investigation, quality of life was measured by the SF-36 Quality of Life Self-Measurement Scale, revealing a positive correlation between wellbeing and quality of life (27). Similarly, in a study of quality of life among adults with paraplegia, it was concluded that quality of life was significantly associated with subjective wellbeing (28).

Many studies have explored factors that influence subjective wellbeing, especially among older adults, such as physical health, mental health, living environment and social phenomena. Meanwhile, studies of overall quality of life have been conducted primarily on patients, such as cancer patients (29), particularly breast cancer patients (30), and adult paraplegics (28), but less so on older adults. Thus, the present study investigated subjective wellbeing and quality of life among older adults, particularly the correlation between them. More specifically, the study explored the influence of quality of life on subjective wellbeing among older adults and thereby provides a reference base for future research aimed at enhancing subjective wellbeing among older adults.

## 2. Methods

### 2.1. Design

This study used a cross-sectional questionnaire to examine the relationship between subjective wellbeing and the quality of life of older adults in the community in Dongguan City. Participants were required to undergo a face-to-face interview with an investigator lasting 20–25 min, during which the trained investigator and participants conversed in the local language. All participants were informed of the study’s purpose beforehand and were assured that their information and responses would remain confidential and anonymous. Participants also signed a written informed consent form.

### 2.2. Subjects

The research was conducted among older adult residents of Dongguan City from December 2018 to February 2019. In this study, ‘older adult’ was defined as individuals no younger than 60 years old and living in the community. An eligible list of older adults for the study was provided by the community committee. A multistage cluster sampling survey technique was used, and 892 older adults were invited to take part in the study (98.4% response rate). In the first stage, four districts were purposively selected out of 33 districts. In the second stage, 22 clusters were randomly selected from 26 communities with a probability proportional to older adults’ density. In the third stage, older adults were selected randomly within each cluster.

### 2.3. Measures

Subjective wellbeing was assessed using the Memorial University of Newfoundland Well-Being Scale (MUNSH) (31), which was widely used in geriatric research to measure the quality of life and health status of older adults. The scale consists of 24 items across four dimensions: five items in positive affect (PA), five items in negative affect (NA), seven items in positive experience (PE), and seven items in negative experience (NE). The scoring method was as follows: 2 points for ‘yes,’ 1 point for ‘do not know,’ and 0 points for ‘no,’ resulting in a total score range of −24 to 24. The scale was employed to assess the subjective wellbeing of Chinese older adults (32, 33). The Cronbach’s α coefficients of the four sub-dimensions of the MUNSH scale in the present study were 0.717, 0.739, 0.693, and 0.713, respectively, with a KMO coefficient of 0.907, indicating good reliability.

Quality of life was measured using the SF-36 Quality of Life Self-Measurement Scale (34), which consists of eight dimensions: 10 items for physical functioning (PF), four items for role-physical (RP), two items for bodily pain (BP), five items for general health (GH), four items for vitality (VT), two items for social functioning (SF), three items for role-emotional (RE), five items for mental health (MH), and one item to evaluate health status in the past year. The total number of entries was 36. Additionally, physical functioning, role-physical, bodily pain and general health were categorized as components of physical health (PCS), while role-physical, social functioning, mental health and vitality were categorized as components of mental health (MCS) – with higher scores indicating better quality of life. The Chinese version of the SF-36 is a valid and reliable tool for assessing health-related quality of life (HRQOL) within the Chinese population (35).

An original general demographic questionnaire was developed to collect data about the participants’ demographic characteristics, health status and social circumstances.

### 2.4. Statistical analysis

The data were organized using SPSS 26.0 software, after which a simple correlation analysis and canonical correlation analysis were performed.

Canonical correlation analysis (36) is a multivariate statistical method used to examine the correlation between two sets of variables. It involves extracting two representative integrated random variables from two groups of variables through dimensionality reduction, and these two variables are referred to as a pair of canonical variables. The correlation observed between the canonical variables is used to reflect the correlation between the original two groups of variables. The first set of variables was denoted as $X={\left(X_{1},\;\ldots ,\;X_{p}\right)}^{\prime }$, while the second set was denoted as $Y={\left(Y_{1},\;\ldots ,\;Y_{q}\right)}^{\prime }$. Canonical correlation analysis extracted canonical components U from the first set of variables X via principal component analysis (U was a linear combination of $X_{1},\;\ldots ,\;X_{p}$). The canonical components V were then extracted from the second set of variables Y (V was a linear combination of $Y_{1},\;\ldots ,\;Y_{q}$) while maximizing the correlation between U and V. This U–V correlation reflected the correlation between the two sets of variables X and Y (37, 38). Canonical loadings analysis generated correlations between the original and canonical variables. Empirically, variables with an absolute value of 0.3 or greater in the canonical loadings were considered to meaningfully explain the canonical variables of interest (39). In this study, we selected important loadings using a cutoff value of 0.35.

Canonical redundancy (40) reflects the ability of each canonical variable to explain all variables in the variable group and can be divided into first and second canonical redundancy depending on the direction of action. First canonical redundancy indicates the percentage of canonical variables explained by their own variable group, whereas second canonical redundancy indicates the percentage of canonical variables explained by another variable group, numerically equal to the product of the first canonical redundancy and the square of the canonical correlation coefficient. The square of the canonical correlation coefficient indicates the percentage of common variation between the two groups of canonical variables.

The scores on the four dimensions – positive affect, negative affect, positive experience and negative experience (Y1 ~ Y4) – of the MUNSH were utilized as canonical variables U, while the eight dimensions – physical functioning, role-physical, bodily pain, general health, vitality, social functioning, role-emotional, and mental health (X1 ~ X8) – of the SF-36 scale were employed as canonical variables V. Canonical correlation analysis was performed between the U variables and the V variables, and a significance level of *α* = 0.05 was applied.

### 2.5. Ethical considerations

Ethical approval was received from the Institutional Ethics Committee of Guangdong Medical University, China (REC: PJ2018037) before the research was conducted. Privacy and data confidentiality were ensured. All participants were fully informed that their participation was entirely voluntary and that they could unconditionally withdraw from the study at any time. A small gift was given to each participant as a token of gratitude for their participation.

## 3. Results

### 3.1. Descriptive material

A total of 892 older adults, aged between 60 to 99 years old, with an average age of 68.592 ± 7.063, participated in the survey. Among the participants, 353 were male and 539 were female, with the majority being married (79.596%). The participants’ education level were distributed as follows: 602 had elementary school education or lower, 174 had middle school education, 96 had high school education, and 20 had college education or higher, as shown in Table 1.

**Table 1:** Results of the analysis of the socio-demographic characteristics of the sample, *n* = 892.

Variables		*n* (%)	Variables		*n* (%)
Gender	Male	353 (39.574)	Marital status	Married/cohabiting	710 (79.596)
	Female	539 (60.426)		Other	182 (20.404)
Age (years)	60 ~ 69	571 (64.013)	Currently working	Yes	87 (9.753)
	70 ~ 79	241 (27.018)		No	805 (90.247)
	≥80	80 (8.969)	Healthcare insurance	Yes	709 (79.484)
Education level	Primary school or below	602 (67.489)		No	183 (20.516)
	Junior High School	174 (19.507)	Self-rated health status	Good	377 (42.265)
	High School	96 (10.762)		Fair	410 (45.964)
	Bachelor’s degree or higher	20 (2.242)		Poor	105 (11.771)

### 3.2. Subjective wellbeing and quality of life scores of older adults

The total score of subjective wellbeing among older adults participants was 13.820 ± 8.975, with the following sub-scores: positive affect (7.418 ± 2.600), negative affect (1.776 ± 2.504), positive experience (10.264 ± 3.333) and negative experience (2.087 ± 2.783). The quality of life scores of the participants were as follows: physical functioning (82.786 ± 18.982), role-physical (71.553 ± 40.324), bodily pain (80.410 ± 19.653), general health (64.209 ± 19.845), vitality (75.471 ± 16.313), social functioning (83.981 ± 18.05), role-emotional (74.439 ± 37.264), and mental health (76.054 ± 15.303) (Table 2).

**Table 2:** Scores of subjective wellbeing and quality of life dimensions among older adults, *n* = 892.

Variables	Possible range	Min	Max	Mean	SD
Positive affect	0–10	0	10	7.418	2.600
Negative affect	0–10	0	10	1.776	2.504
Positive experience	0–14	0	14	10.264	3.333
Negative experience	0–14	0	14	2.087	2.783
Total (subjective wellbeing)	–24 – 24	−21	24	13.820	8.975
Physical functioning	0–100	0	100	82.786	18.982
Role-physical	0–100	0	100	71.553	40.324
Bodily pain	0–100	12	100	80.410	19.653
General health	0–100	0	100	64.209	19.845
Vitality	0–100	5	100	75.471	16.313
Social functioning	0–100	11	100	83.981	18.05
Role-emotional	0–100	0	100	74.439	37.264
Mental health	0–100	16	100	76.054	15.303

### 3.3. Simple correlation analysis

Pearson’s simple correlation analysis was performed on the dimensions of both quality of life and subjective wellbeing. The results showed that the positive affect, positive experience and total subjective wellbeing scores were significantly and positively correlated with each of the quality of life dimensions, whereas negative affect and negative experience were significantly and negatively correlated with each of the quality of life dimensions (Table 3).

**Table 3:** Simple correlation analysis of subjective wellbeing scores and quality of life scores of older adults, *n* = 892.

Variables	Positive affect	Negative affect	Positive experience	Negative experience	Total (subjective wellbeing)
Physical functioning	0.282^**^	−0.264^**^	0.344^**^	−0.316^**^	0.381^**^
Role-physical	0.245^**^	−0.242^**^	0.245^**^	−0.276^**^	0.315^**^
Bodily pain	0.310^**^	−0.311^**^	0.286^**^	−0.296^**^	0.375^**^
General health	0.420^**^	−0.368^**^	0.421^**^	−0.352^**^	0.490^**^
Vitality	0.493^**^	−0.461^**^	0.476^**^	−0.540^**^	0.616^**^
Social functioning	0.331^**^	−0.299^**^	0.337^**^	−0.340^**^	0.410^**^
Role-emotional	0.175^**^	−0.266^**^	0.195^**^	−0.272^**^	0.282^**^
Mental health	0.480^**^	−0.451^**^	0.410^**^	−0.510^**^	0.576^**^

The value in the table represents the correlation coefficient, **Means that the correlation is significant when the confidence level (two-sided) is 0.001.

### 3.4. Canonical correlation analysis

#### 3.4.1. Canonical correlations and the tests

The canonical correlation analysis extracted a total of four pairs of canonical variables, all of which had statistically significant correlation coefficients (0.695, 0.179, 0.147, 0.121) (*p* < 0.05). Given the high cumulative contribution of the first pair of canonical variables, which accounted for 93.030%, further interpretation was provided for this pair, as shown in Table 4.

**Table 4:** Canonical correlation coefficients between wellbeing and QOL in older adults.

Canonical pairs	Correlation coefficient	Eigenvalue	Cumulative proportion%	Wilk’s	Approximate *F* value	*P-*value
1	0.695	0.934	93.030	0.483	22.163	<0.001
2	0.179	0.033	96.315	0.933	2.933	<0.001
3	0.147	0.022	98.506	0.964	2.720	0.001
4	0.121	0.015	100.00	0.985	2.619	0.023

#### 3.4.2. Standardized canonical coefficients for canonical variables

The magnitude of the absolute value of the standardized coefficients in the canonical correlation analysis represents the magnitude of the weights. As indicated by the standardized first canonical correlation coefficients (Table 5), for the first canonical variate (U1), the standardized canonical coefficients of physical functioning, role-physical, bodily pain, general health, vitality, social functioning, role-emotional, and mental health, as the variables of quality of life, were − 0.128, −0.010, −0.022, −0.222, −0.442, −0.007, 0.031, and −0.447, respectively. The coefficients of vitality (−0.442) and mental health (−0.447) contributed the most to quality of life. For the first canonical variate (V1), positive affect, negative affect, positive experience and negative experience were the variables of subjective wellbeing, and the standardized canonical coefficients were −0.338, 0.225, −0.292, and 0.388, respectively. The standardized canonical coefficients were approximately equal in absolute magnitude, indicating that the first canonical variate (V1) was reflected by a combination of positive affect (−0.338), negative affect (0.225), positive experience (−0.292) and negative experience (0.388). The two formulas presented below were derived from the canonical correlation analysis.

**Table 5:** Coefficients of standardized canonical variables for quality of life and subjective wellbeing of older adults, *n* = 892.

Quality of life variable set		Subjective wellbeing variable set	
Variables	U1	Variables	V1
Physical functioning (X1)	−0.128	Positive affect (Y1)	−0.338
Role-physical (X2)	−0.010	Negative affect (Y2)	0.225
Bodily pain (X3)	−0.022	Positive experience (Y3)	−0.292
General health (X4)	−0.222	Negative experience (Y4)	0.388
Vitality (X5)	−0.442		
Social functioning (X6)	−0.007		
Role-emotional (X7)	0.031		
Mental health (X8)	−0.447		

These equations are as follows:


$$\begin{array}{l} X=-0.128X_{1}-0.010X_{2}-0.022X_{3}-0.222X_{4}-0.442X_{5}\\ \;-0.007X_{6}+0.031X_{7}-0.447X_{8} \end{array}$$

$$Y=-0.338Y_{1}+0.225Y_{2}-0.292Y_{3}+0.388Y_{4}$$

#### 3.4.3. Canonical loadings and cross loadings

Due to the large correlation between the dimensions of quality of life and those of subjective wellbeing as well as the possibility of covariance, explaining the problem solely through canonical weights would have been insufficient. In order to better reflect the relationship between the original and canonical variables, their correlation required further analysis by canonical loadings and cross-loadings (36).

As shown by the first set of canonical loading coefficients (Table 6), according to the canonical loading results, for the first pair of canonical variables of quality of life (U1), the most effective factor was vitality (−0.891), followed by mental health (−0.837), general health (−0.696), social functioning (−0.589), physical functioning (−0.543), bodily pain (−0.537), role-physical (−0.455) and role-emotional (−0.405). On the other hand, it showed that the first pair of canonical variables of quality of life (U1) was negatively correlated with the eight dimensions of quality of life. Similarly, for the first pair of canonical variables of subjective wellbeing (V1), the most effective factor was negative experience (0.848), followed by positive affect (−0.817), negative affect (0.755) and positive experience (−0.722). On the other hand, it demonstrated that the first pair of canonical variables of subjective wellbeing (V1) was negatively related to positive affect and positive experience, and positively related to negative affect and negative experience. Combining the canonical correlation coefficients revealed that the dimensions of quality of life were positively correlated with positive affect and positive experience of subjective wellbeing, and negatively correlated with negative affect and negative experience of subjective wellbeing.

**Table 6:** The loadings and cross loadings of the variables for the first canonical function in canonical correlation analysis, *n* = 892.

Quality of life variable set			Subjective wellbeing variable set		
Variables	Loadings	Cross loadings	Variables	Loadings	Cross loadings
Physical functioning (X1)	−0.543	−0.378	Positive affect (Y1)	−0.817	−0.568
Role-physical (X2)	−0.455	−0.316	Negative affect (Y2)	0.755	0.524
Bodily pain (X3)	−0.537	−0.373	Positive experience (Y3)	−0.772	−0.537
General health (X4)	−0.696	−0.484	Negative experience (Y4)	0.848	0.589
Vitality (X5)	−0.891	−0.619			
Social functioning (X6)	−0.589	−0.409			
Role-emotional (X7)	−0.405	−0.281			
Mental health (X8)	−0.837	−0.581			

According to the cross-loadings, physical functioning, bodily pain, general health, vitality, social functioning and mental health were strongly correlated with subjective wellbeing (absolute value of cross-loadings was greater than 0.35), with cross-loadings of –0.378, −0.373, −0.484, −0.619, −0.409, and −0.581, respectively. Physical functioning, bodily pain, general health, vitality, social functioning and mental health were found to be the main factors reflecting subjective wellbeing among older adults.

#### 3.4.4. Redundancy analysis

As can be seen from Table 7, the first canonical redundancy of the quality of life variable was 0.638, whereas the second was 0.308, indicating that the cumulative percentage of quality of life differences explained by their own canonical variables was 63.8%, while the cumulative percentage of subjective wellbeing differences explained by paired canonical variables was 30.8%. Similarly, the cumulative percentage of subjective wellbeing explained by its own canonical variables was 41.0%, whereas the cumulative percentage of quality of life explained by paired canonical variables was 19.8%. This demonstrates that quality of life and subjective wellbeing can be explained not only by their own canonical variables but also by each other’s canonical variables. The square of the canonical correlation coefficient was 0.483, indicating that the first canonical variable explained a certain proportion of the shared variance, further verifying the reliability of the analytical results.

**Table 7:** Explanatory ability of canonical variables.

Canonical variable	First canonical redundancy (*R*_1_^2^)	Square of canonical correlation coefficient (*R*_c_^2^)	Second canonical redundancy (*R*_2_^2^)
U	0.638	0.483	0.308
V	0.410	0.483	0.198

## 4. Discussion

With the growing interest in improving the subjective wellbeing of older adults, it was important to understand the relationship between subjective wellbeing and quality of life. From the simple correlation analysis, it was clear that the quality of life of older adults was positively correlated with the total score of subjective wellbeing – importantly, the higher the quality of life of an older adult, the higher their subjective wellbeing, which was consistent with the findings of Bachmann et al. (41). This was attributable to the fact that older adults who had a high quality of life were more often in good health, while older adults with good subjective wellbeing also tended to have a healthy psyche and positive emotions (42). On the other hand, older adults with a good quality of life were less prone to negative emotions and had a higher level of life satisfaction (32, 33), thus leading to a higher level of subjective wellbeing.

Canonical correlation analysis showed that the dimensions of quality of life in older adults were positively correlated with positive affect and positive experience, and negatively correlated with negative affect and negative experience. Higher levels of physical function, role-physical, bodily pain and general health, reflecting somatic health in older adults, were associated with higher levels of the positive affect and positive experience, and lower levels of the negative affect and negative experience. This finding suggests that somatic health may have an effect on subjective wellbeing (37, 38). Physical health directly affected older adults’ experience of normal life. The healthier older adults were, the more energetic they felt, the more they interacted with others, the less lonely and miserable they felt, and the higher their subjective wellbeing. When older adults’ physical health was optimal, they had a greater likelihood of experiencing joy in life, which in turn enhanced their positive emotions and positive experiences of subjective wellbeing while minimizing their negative emotions and negative experiences to a corresponding degree (43). Conversely, poorer physical health due to chronic illness, painful experiences and/or economic stress all increased psychological stress in older adults, leading to anxiety and depression, which in turn directly affected their subjective wellbeing (44).

Similarly, the higher the vitality, social functioning, emotional functioning and mental health, all of which reflected the mental health of older adults, the higher the positive affect and positive experience, and the lower the negative affect and negative experience. Older adults might also have been more likely to experience loneliness due to a potential lack of family and friends (45). Negative affect was exacerbated among older adults who experienced prolonged feelings of sadness or depression. This, in turn, affected their perception estimation of life satisfaction and subjective wellbeing.

Canonical redundancy analysis was used to reflect the degree of variance explained by each typical variable with respect to the original set of variables as a whole. From the above results, it was clear that the subjective wellbeing of older adults could not be fully reflected in terms of quality of life, as it was also influenced by other factors. Therefore, when examining the subjective wellbeing of older adults, various factors needed to be comprehensively considered.

A correlation was observed between the quality of life and subjective wellbeing of older adults. Consequently, it is important to take appropriate measures that consider the individual characteristics of older adults to address their physical and psychological challenges. This approach can help older adults gain a more accurate perception of their quality of life and assess their subjective wellbeing. Moreover, it can mitigate the adverse effects of physical and psychological issues on their quality of life and emotional state, thereby enhancing not only their quality of life but also their subjective wellbeing. Due to the natural process of aging, degenerative changes in cognitive functions among older adults are inevitable and ultimately affect their physical and psychological health. Consequently, older adults are more susceptible to experiencing negative emotions and mental health problems. Therefore, it becomes crucial for family members, friends, and the community at large to closely monitor the psychological wellbeing of older adults. This vigilance can help identify negative factors at an early stage and facilitate constructive communication with older adults to address these factors before they escalate. Older adults should strive to maintain a positive and optimistic attitude, embracing every day with a positive mindset, and pursuing ‘happiness in old age.’

This study was limited by the available manpower and materials, and it was therefore not possible to conduct more extensive surveys in other areas of Dongguan City. In addition, subjective wellbeing among older adults is affected not only by quality of life but also by leisure and recreation, social environment, etc. Therefore, more substantial research is needed on the factors affecting subjective wellbeing among older adults. It is expected that, in the future, more in-depth research will be performed with more rigorous measures in the interest of improving the subjective wellbeing of older adults and helping them to age gracefully.

## Data availability statement

The raw data supporting the conclusions of this article will be made available by the authors, without undue reservation.

## Ethics statement

The study was conducted in accordance with the Declaration of Helsinki, and approved by the Institutional Ethics Committee of Guangdong Medical University, China (REC: PJ2018037). Written informed consent was obtained from all subjects involved in the study.

## Author contributions

HL, QG, and YL participated in study design, data collection, data analysis, and manuscript writing. QG, JT, and XS contributed to study design and made comments on the manuscript. CW assisted in writing the materials, collecting the data, and commenting on the manuscript. CW and YL was responsible for the overall content as the guarantor. All authors contributed to the article and approved the submitted version.
